# Studies on the electrostatic effects of stretched PVDF films and nanofibers

**DOI:** 10.1186/s11671-021-03536-9

**Published:** 2021-05-03

**Authors:** Yixuan Lin, Yuqiong Zhang, Fan Zhang, Meining Zhang, Dalong Li, Gaofeng Deng, Li Guan, Mingdong Dong

**Affiliations:** 1 Department of Chemistry, Renmin University of China, Beijing, 100872 People’s Republic of China; 2 School of Marine Science and Technology, Harbin Institute of Technology At Weihai, Weihai, 264209 Shandong People’s Republic of China; 3 Sino-Danish Center for Education and Research (SDC), Interdisciplinary Nanoscience Center (iNANO), Aarhus University, DK-8000 Aarhus C, Denmark; 4 State Key Laboratory of Building Safety and Environment, China Academy of Building Research, Beijing, 100013 People’s Republic of China

**Keywords:** Β-phase PVDF, Mechanical stretching, Electrospinning, Pyro- and piezoelectric effects

## Abstract

The electroactive β-phase in Poly (vinylidene fluoride, PVDF) is the most desirable conformation due to its highest pyro- and piezoelectric properties, which make it feasible to be used as flexible sensors, wearable electronics, and energy harvesters etc. In this study, we successfully developed a method to obtain high-content β-phase PVDF films and nanofiber meshes by mechanical stretching and electric spinning. The phase transition process and pyro- and piezoelectric effects of stretched films and nanofiber meshes were characterized by monitoring the polarized light microscopy (PLM) images, outputting currents and open-circuit voltages respectively, which were proved to be closely related to stretching ratio (*λ*) and concentrations. This study could expand a new route for the easy fabrication and wide application of PVDF films or fibers in wearable electronics, sensors, and energy harvesting devices.

**Supplementary Information:**

The online version contains supplementary material available at 10.1186/s11671-021-03536-9.

## Introduction

Over the past decades, different kinds of electrostatic materials such as inorganic ceramics, pyro- or piezoelectric polymers, and composite-based materials have been investigated and widely applicated in nanogenerators and flexible devices etc. Some inorganic electrostatic materials, such as BaTiO_3_, PZT, and PbTiO_3_ etc., have been used in many fields, which were reported with toxicity, high costs, and possible pollution to the environment. Compared with those lead-based pyro- or piezoelectric materials, organic polymers such as polyvinylidene fluoride (PVDF), polyacrylonitrile (PAN) etc. have good flexibility, excellent insulation and machinability. These properties make them feasible to be adopted in nanogenerators [1, 2], flexible sensors [3, 4], energy harvesters [5, 6] and so on. Among these pyro- and piezoelectric polymers, PVDF has been widely used in many fields due to its high dielectric constant, high-energy storage density, and well chemical stability. In 1960s, PVDF, a polymer material which could have strong piezoelectric effect after treating with high-temperature, strong electric field polarization, or uniaxial stretching was first discovered by Kawai [7]. Later, Bergmant et al. treated PVDF with electric field polarization and mechanical stretching, and found that it also has electrostatic effects [8–11]. PVDF film has been widely applicated in the fields of sensing [12–14], oil water separation [15–17], antifouling and antibacterial membrane [18–20], and biological membrane [21–23] based on its pyro- and piezoelectric effects [24].

Depending on its different chain conformations of trans (T) and gauge (G), there are five crystal phases (α, β, γ, δ, and ε) of PVDF [25–27]. The α-phase (TGTG) is the most stable phase and most of them can be obtained by isothermal crystal phase without any treatment [28–30]. β-phase (TTTT) is the phase that exhibits spontaneous polarization strength and pyro- and piezoelectric properties, because the fluorine atoms in the β-phase are located on the same side of the molecular chains, which are arranged parallel to each other in a specific direction, with the same dipole orientation and enhanced polarity [31–33]. Since the β-phase has pyro- and piezoelectric effects, but α-phase does not, when the PVDF conformation transfers from α-phase to β-phase with dipoles, the polymers exhibit pyro- and piezoelectric capabilities. Therefore, we need to convert the α-phase to β-phase by some methods.

A series of modification methods, such as electric field polarization [34], supercooled crystallization [35], co-crystallization [36, 37], and restricted crystallization [38] are adopted to obtain the β-phase. Electric field polarization is a method in which a non-uniform electric field in an atmospheric atmosphere causes a partial breakdown of the air by a corona discharge, resulting in ion beam bombardment of the dielectric and deposition of ionic charges in the dielectric to form a laterally uniformly distributed piezoelectric ferrite of high charge density. However, since the excitation of air-gap ions by the electric field is very limited, the charge can only be deposited on and near the surface of the sample. Crystallization method is a process in which the static amorphous polymer system is obtained by evaporating solvent. In the crystallization method, the solvent polarity, solution concentration, evaporation rate, and other factors can affect the crystalline phase of PVDF, making it difficult to control the experimental conditions. Hence, considering the effect of solvents on crystallization, we need a simple and fast method to prepare PVDF films that eliminate solvents.

In this study, the mechanical stretching method was adopted to obtain the β-phase PVDF film with the advantages of convenient preparation and rapid prototyping [39–41]. We report our experimental observations through a temperature assisted stretching processing to achieve phase transition and pyro- and piezoelectric effects of PVDF films. A polarized light microscopy (PLM) was adopted to monitor the phase transfer processing, which allows rapid and intuitive observations of the surface topographies, preliminary determinations of the surface structure of the samples, and assessment of the crystallinity of the organic films [42–44]. The FTIR, XRD, and Raman further characterized the phase distribution of the stretched PVDF. The pyro- and piezoelectric effects were characterized by an electrochemical workstation. Furthermore, PVDF nanofiber meshes were successfully fabricated by electrostatic spinning. The stretching process during the spinning could facilitate the form of the β-phase, and hence the pyro- and piezoelectric effects.

## Materials and methods

The PVDF powders (Solvay, USA) were commercially available with an average molecular weight ~ 640,000. The solvent N,N-Dimethylformamide (DMF) was purchased from Beijing Chemical Works, and ethyl acetate was purchased from Beijing TongGuang Fine chemicals Company. All of these materials and solvents were used as received without further purification.

### Fabrication of PVDF films

The mixed solution of ethyl acetate and DMF with a weight ratio of 6:4 was used to dissolve PVDF powders. The prepared PVDF solutions with different mass fraction (6 wt%, 8 wt%, 10 wt% 11 wt%, 12 wt%, 13 wt%) were spin-coated on silicone substrates to obtain PVDF films by KW-4A. The films were spin-coated under a rotational speed of 2000 rpm for 15 s. Then the prepared PVDF membrane with a thickness of 700 nm (Additional file 1: Figure S1), which was tested by Profilometer, was uniformly stretched under 80 °C at a stretching rate of 10 μm/s by Linkam TST350.

### Fabrication of PVDF nanofiber meshes

The polymer solution was loaded into a syringe, which was connected by a metal nozzle with an inner diameter of 0.65 mm. Then the solution was electrospun into nanofibers and collected on a non-woven fabric. The parameters of the electrospinning were set as following: the distance between the spinneret and the collector was 15 cm, the high-voltage power supply was 15 kV, the volume feed rate was 0.5 mL/h, which was subjected by air pressure, respectively, the range of humidity is 10–40% RH at 25 °C.

### Characterization

The surface morphologies of the PVDF film were characterized by a scanning electron microscope (SU8010, HITACHI). The crystal structures of the PVDF film were characterized by Fourier transform infrared spectrometer (FTIR, TENSOR 27, BRUKER), Raman spectrometer (HORIBA T64000), and X-ray diffraction (XRD 7000, Shimadzu). A Polarized Light Microscopy (PLM, Zeiss Axio Scope.A1) characterized the conformations of PVDF films during stretching. A DC supplier (Keithley 2410 SourceMeter) was used to provide variable voltages to the motor and heat plate, so that the composite film sensor closely adhered to the heater chips could work under different frequencies and temperatures. The fabricated PVDF device were connected to an electrochemical workstation (CHI660D, Shanghai Chenhua Instrument Co., Ltd.) to characterize the pyro- and piezoelectric effects. The real-time current signals under different frequencies and temperatures were monitored by using the chronoamperometry method of the electrochemical workstation analyzer. The parameters during the measurements were: Init E 0 V, Sample Interval 0.001 s^−1^.

## Results and discussion

**Fig. 1:**
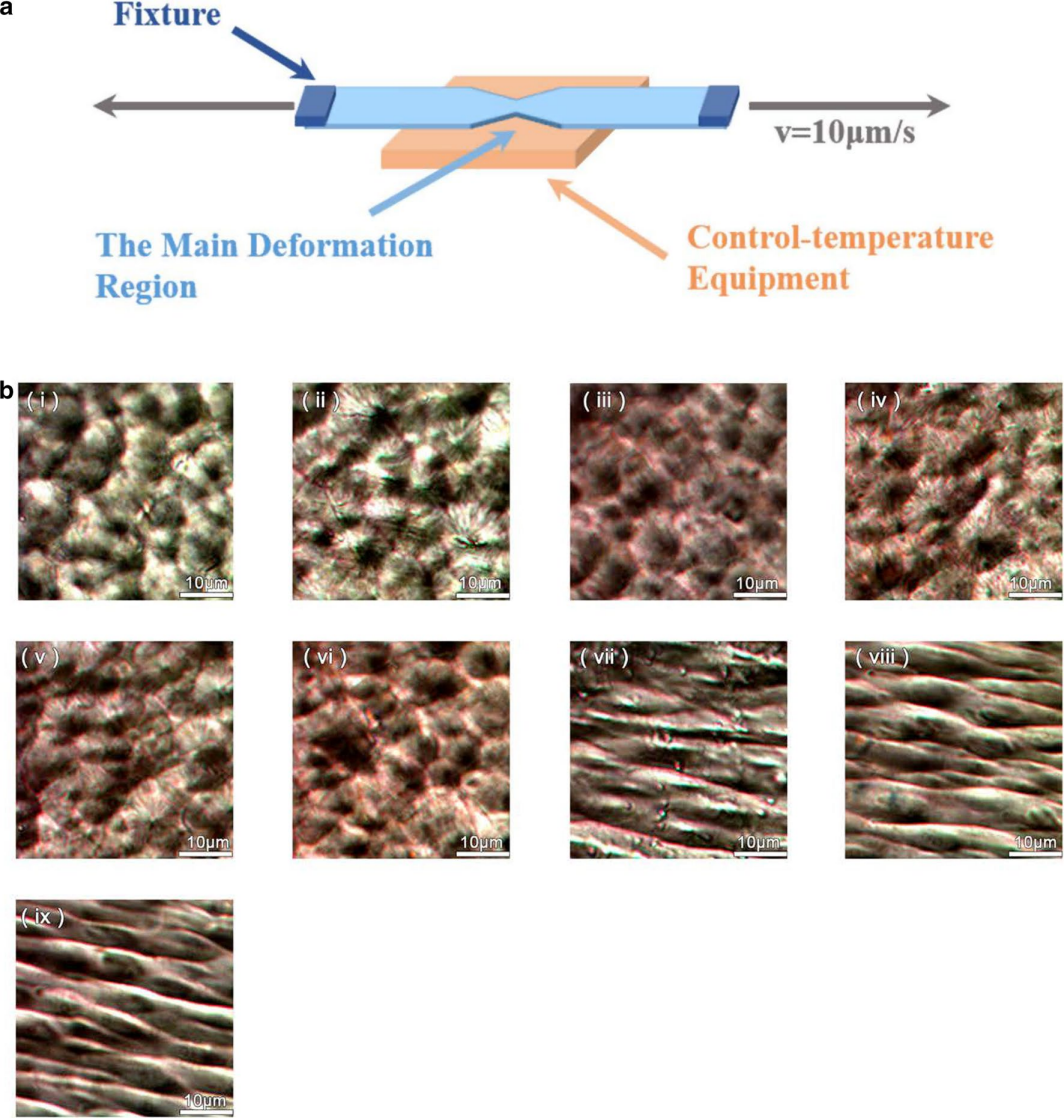
**a** Schematic diagram of the experimental setup for stretching of PVDF film; **b** The PLM images of PVDF films during the stretching with different tensile ratios, *λ* = 1 (**i**), *λ* = 1.02 (**ii**), *λ* = 1.04 (**iii**), *λ* = 1.06 (**iv**), *λ* = 1.08 (**v**), *λ* = 1.1 (v**i**), *λ* = 1.2 (**vii**), *λ* = 1.3 (v**iii**), *λ* = 1.4 (**ix**)

The conformation of PVDF chain, which was gradually transfer from α-phase with Trans-Gauche-Trans-Gauche (TGTG) into β-phase with Trans–Trans (TT) conformation during stretching, was characterized by a PLM. In order to obtain a uniformly stretched film during the unidirectional stretching, Linkam TST350 was used to stretch the PVDF film, and the moderate temperature 80 °C and a relatively slow stretching rate 10 μm/s were adopted. The schematic diagram was shown in Fig. 1a. With the increase of stretching ratio (*λ*), the crystal phases of the PVDF underwent a significant transformation, changed of crystal shape from spherical to woven, and finally transformed into the β-phase at *λ* = 1.3. The corresponding PLM images during the stretching were shown in Fig. 1b. Accordingly, it could be concluded that at *λ* = 1.3 the α-phase transforms into the β-phase.

**Fig. 2:**
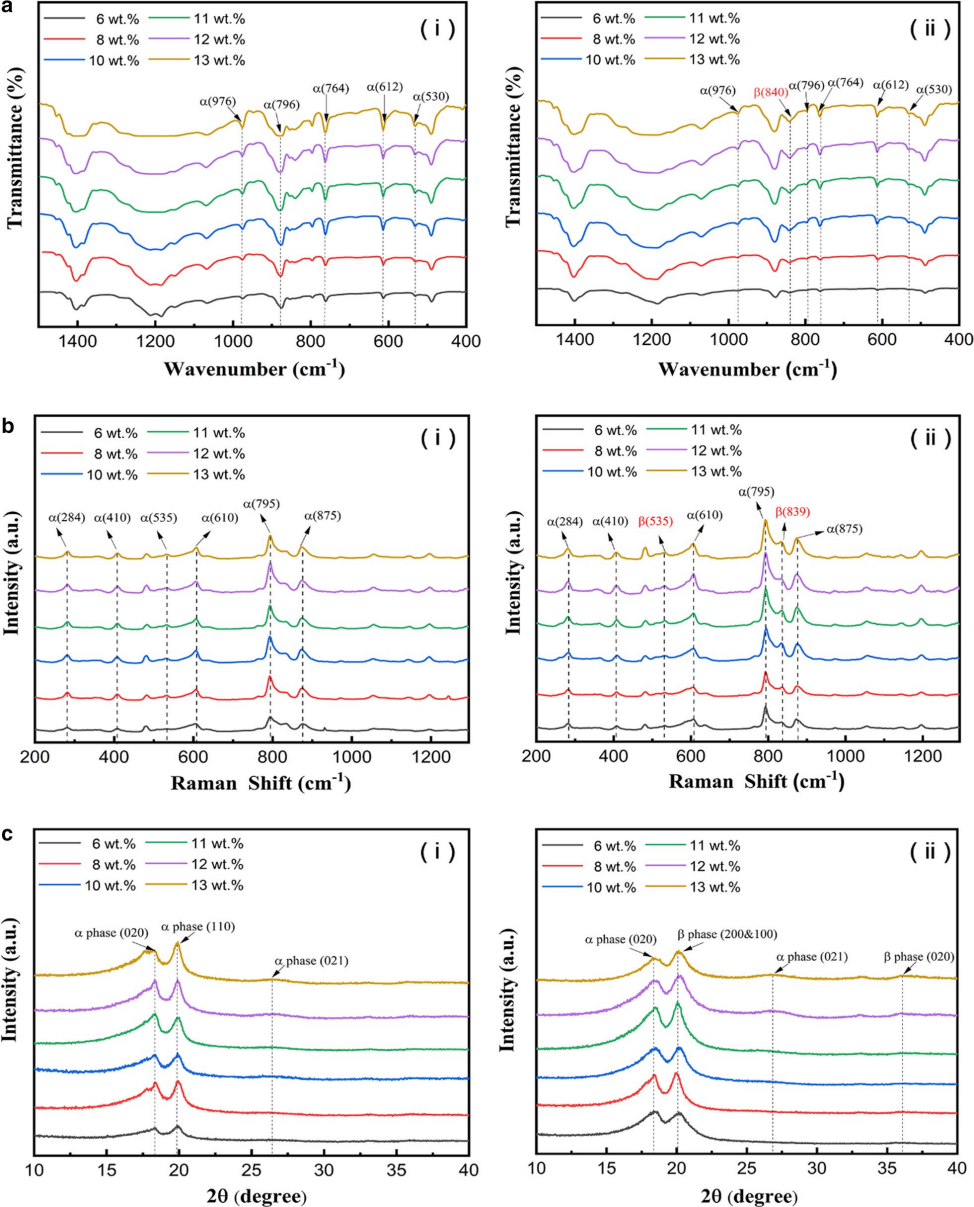
Crystal characterization of PVDF films. **a** FTIR of PVDF films with different mass fractions, original (**i**), stretched (**ii**). **b** Raman of PVDF films with different mass fractions, original (**i**), stretched (**ii**)**. c** XRD of PVDF films with different mass fractions, original (**i**), stretched (**ii**)

A series of characterizations were performed to confirm that the β-phase was indeed produced by stretching. Infrared spectra was achieved using a Fourier Transform Infra-Red (FTIR) spectrophotometer in the wave number range of 400–1500 cm^−1^. The FTIR absorption spectra analysis showed that the PVDF film with α-phase has distinct characteristic absorption peaks at 1383 cm^−1^, 976 cm^−1^, 853 cm^−1^, 796 cm^−1^, 764 cm^−1^, 612 cm^−1^, and 530 cm^−1^ [14, 45, 46], while PVDF with β-phase has distinct characteristic absorption peaks at 1278 cm^−1^, 840 cm^−1^, and 510 cm^−1^ [40, 47]. The FTIR significant characteristic absorption peaks of PVDF films before and after stretching were shown in Fig. 2a. According to Fig. 2a(i), significant characteristic absorption peaks appeared at 976 cm^−1^, 796 cm^−1^, 764 cm^−1^, 612 cm^−1^, and 530 cm^−1^, which were typical α-phase absorption peaks. It demonstrated that the crystal phase of the PVDF before stretching was mainly α-phase. In Fig. 2a(ii), the absorption peak of the β-phase appeared at 840 cm^−1^, and the peaks of the α-phase absorption were weaker. Therefore, it could be concluded that after stretching, the phase in the PVDF film was transformed.

Assuming that IR absorption follows the Lambert–Beer law [48], the *A* absorbance is given by

1 $$A = \log \left( {\frac{I}{{I_{0} }}} \right) = KCXL$$

where *K* is the absorption coefficient at the respective wave number, *L* is the thickness of samples, *C* is the average total monomer concentration, *X* is the degree of crystallinity of each phase, and *I* and *I*_0_ are the transmitted and incident intensity radiations respectively. Since then, the Eq. 2 can be used to calculate the content of β-phase in a system. Detailed data was shown in Additional file 1: Fig. S2 in supporting information.

2 $$F_{\left( \beta \right)} = \frac{{X_{\beta } }}{{X_{\alpha } + X_{\beta } }} = \frac{{A_{\beta } }}{{\left( {\frac{{K_{\beta } }}{{K_{\alpha } }}} \right)A_{\alpha } + A_{\beta } }} = \frac{{A_{\beta } }}{{1.26A_{\alpha } + A_{\beta } }}$$

The Raman spectra before and after stretching of PVDF films were shown in Fig. 2b, the typical α-phase peaks of PVDF film appear at 284 cm^−1^, 410 cm^−1^, 535 cm^−1^, 610 cm^−1^, 795 cm^−1^, and 875 cm^−1^ and the β-phase peaks at 510 cm^−1^ and 839 cm^−1^ respectively [47, 49]. The results showed that the conformation of PVDF chain gradually transferred from α-phase with Trans-Gauche-Trans-Gauche (TGTG) into β-phase with Trans–Trans (TT) conformation (hydrogen and fluorine atoms on the opposite sides of PVDF backbone) after stretching. The XRD characterizations of the PVDF film before and after stretching were shown in Fig. 2c. Untreated PVDF exhibits major crystalline peaks at 18.4°, 20.0°, and 26.5°, assigned to (100), (110), and (021) crystal planes respectively, since nonpolar TGTG conformation of α-phase was present in the untreated PVDF film [49, 50]. In the stretched PVDF films, peaks 18.4° and 26.5° were totally missing and only one peak at 20.6° is present, assigned to (110) and (200) crystal planes, indicating the formation of a pure β-phase structure. PVDF films with these dipoles could be pyro- and piezoelectrically active. Its charging performances and output voltage/current curves benefited in using as pyro- and piezoelectric polymer sensors, nanogenerators, transducers, and other electrical applications.

**Fig. 3:**
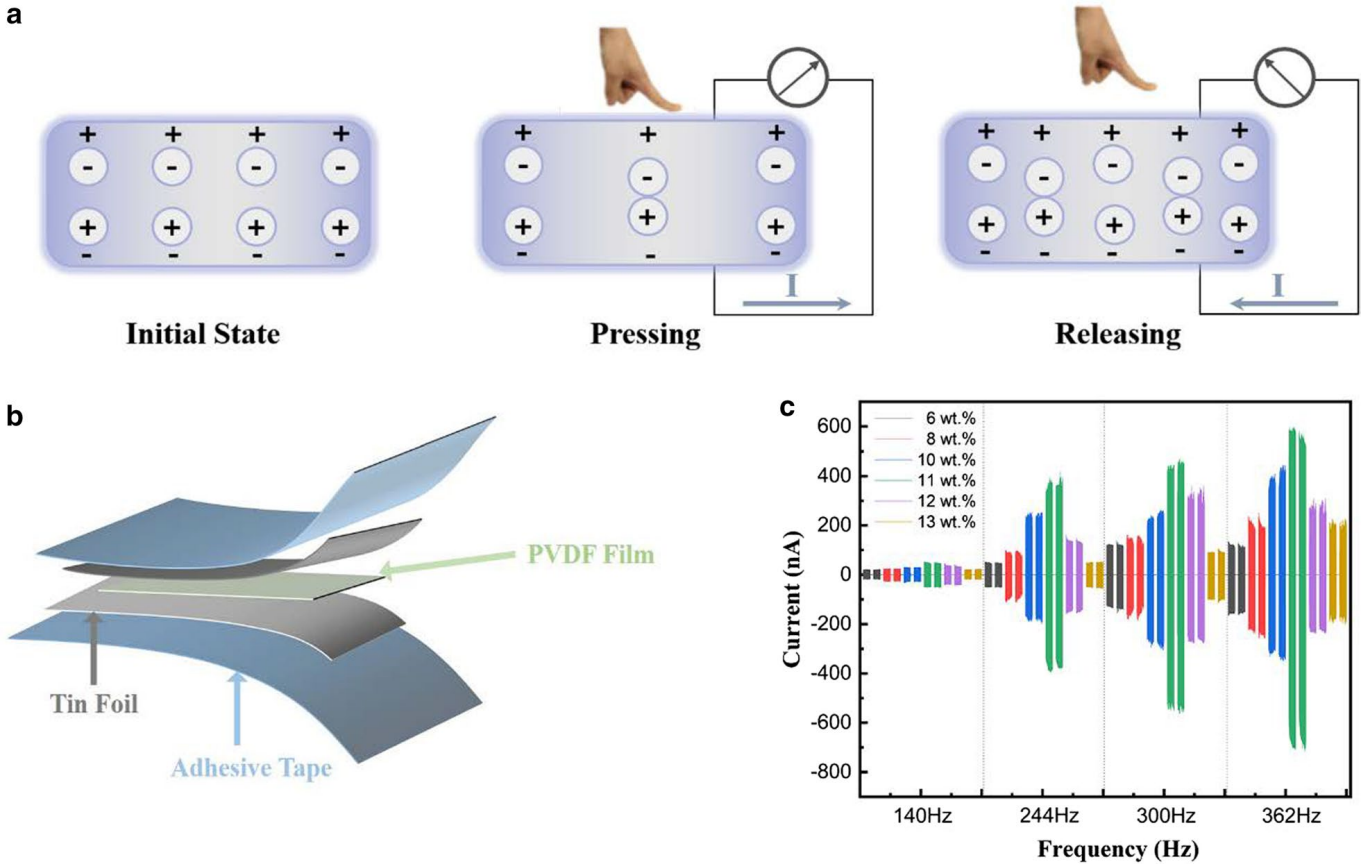
Piezoelectric effects of stretched PVDF films. **a** Schematic diagram of the piezoelectric mechanism under the action of an external force. **b** Schematic diagram of the structures of fabricated PVDF thin film device. **c** The piezoelectric effect of stretched PVDF films (*λ* = 1.3)

Positive piezoelectric effect refers to that the internal polarization of the material will occur with a deformation under the action of an external force, and equal amount of opposite charge will be generated on the two opposite surfaces. When the external force removes, the dielectric material itself will return to the initial states. The mechanism diagram was shown in Fig. 3a. In order to characterize the electrostatic effects of PVDF, a small device with PVDF film was designed and successfully fabricated as Fig. 3b. The piezoelectric currents were monitored by using the pre-designed circuit, when a normal force was applied on the device with repeating press and release cycles. Then the polarization and charge displacement would regulate the piezoelectric charges on the surfaces of device, resulting in the external circuit from the bottom electrode to the top electrode and generating an obvious output current signal. The piezoelectric currents of stretched PVDF films (*λ* = 1.3) at different frequencies were monitored by a motor driven under different voltages (which were supplied by a DC supplier). The results indicated that the output piezoelectric current increased with the increase of mass fractions of PVDF film at the same frequency. The output current reached the maximum when the PVDF concentration was approaching 11 wt%, with a maximum value of 600 nA.

**Fig. 4:**
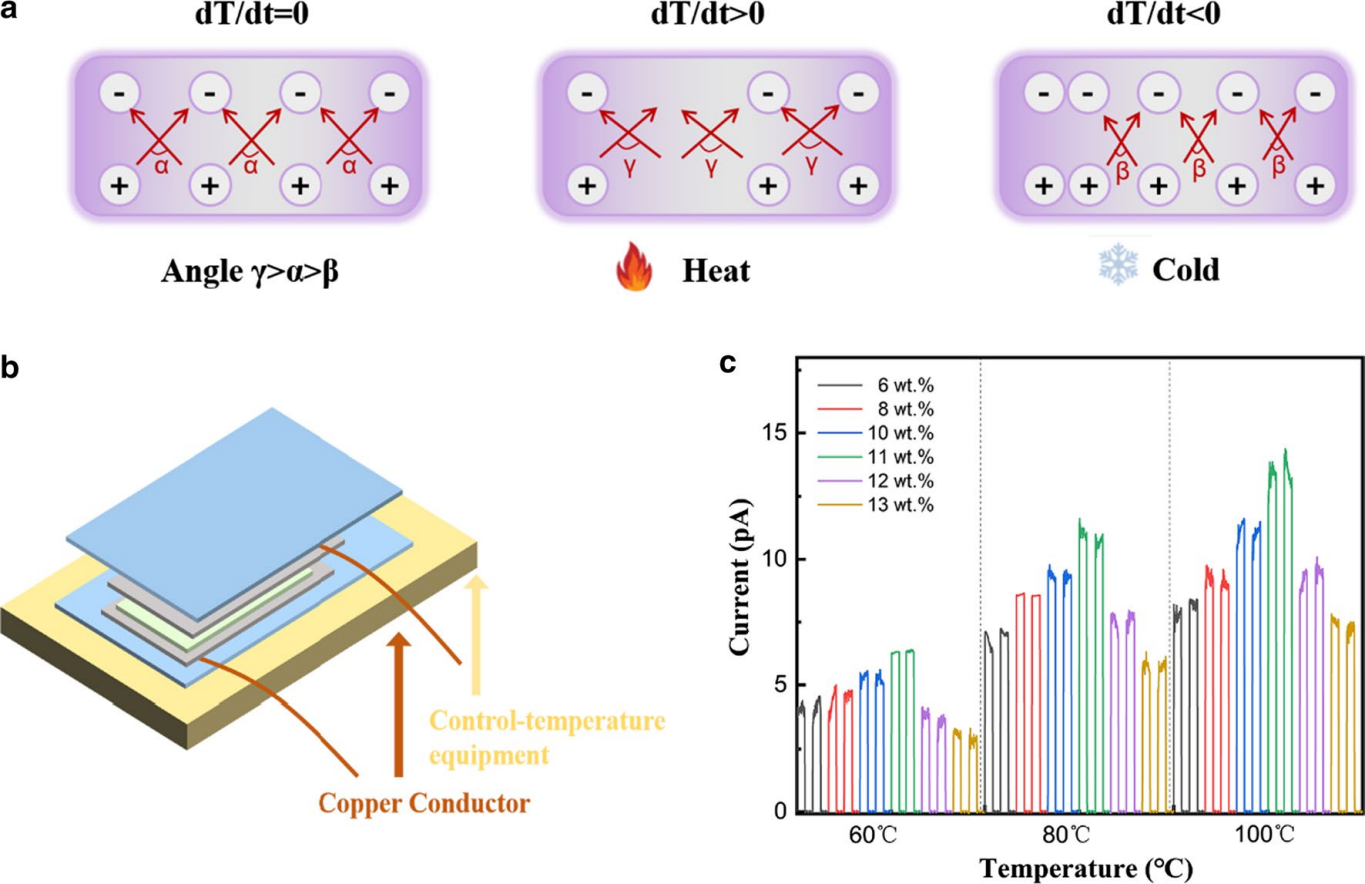
Pyroelectric effects of PVDF films. **a** Schematic diagram of the pyroelectric working mechanism under temperature stimulation; **b** Schematic diagram of fabricated PVDF thin film device; **c** Measurement of pyroelectric effect in PVDF films

Pyroelectric materials can exhibit spontaneous polarization, resulting in positive and negative charges on the film surface with temperature changes. The spontaneous polarization of PVDF films can be changed by heating or cooling at Curie temperature, and electrostatic charges can be generated on both sides of the film. The schematic diagram was shown in Fig. 4a. The pyroelectric effects of PVDF films with different mass fractions were monitored under different temperatures (from 60 to 100 °C) by using a heat plate connected to a DC supplier as shown in Fig. 4b. It could be observed that the output of pyroelectric currents increased with the increase of temperature and reached a maximum value of 15 pA at 100 °C. Similar to the piezoelectric effects, the pyroelectric currents increased with increasing of the mass fractions of PVDF films under the same temperature. The output current reached the maximum when the PVDF concentration approaching 11 wt%, which was consistent with the that of piezoelectric effects, indicating that the concentration of 11 wt% of PVDF films was the most suitable concentration. All these results demonstrated that the PVDF thin film processes excellent pyro- and piezoelectric effects.

**Fig. 5:**
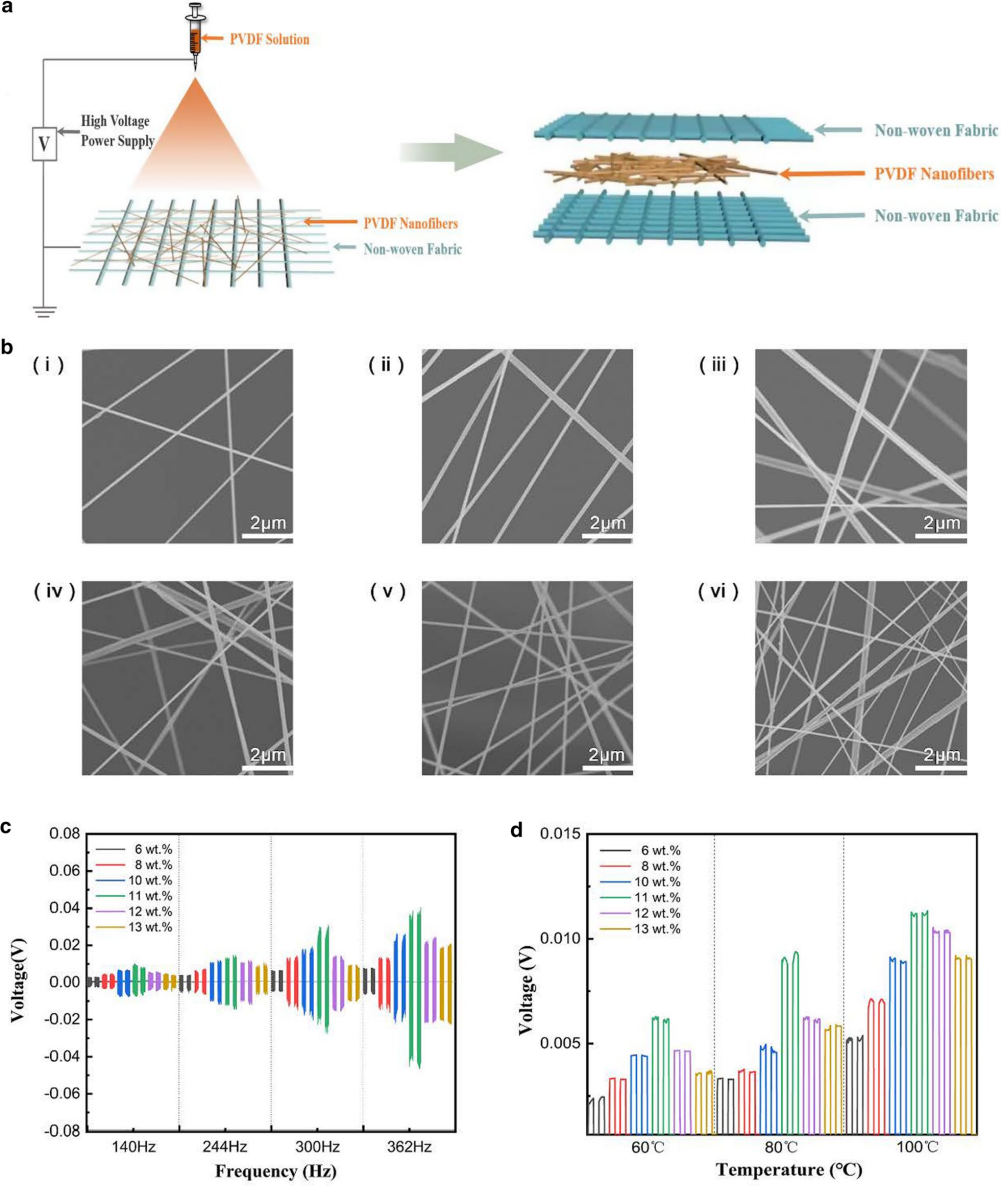
Fabrications and properties of PVDF nanofiber meshes. **a** Schematic diagram of the preparation process of electrostatic spinning. **b** SEM images of PVDF nanofiber`s under different mass fractions: 6 wt% (**i**); 8 wt% (**ii**); 10 wt% (**iii**); 11 wt% (**iv**); 12 wt% (**v**); and13 wt% (**vi**). **c** Piezoelectric effect of PVDF nanofiber mesh with different mass fractions. **d** Pyroelectric effect of PVDF nanofiber mesh with different mass fractions

Due to the distinct electrostatic effect, PVDF could act as potential air-filtering material by adsorption of atmospheric particulates. To explore the air-filtering applications of the PVDF, we fabricated a sandwich structured nanofiber mesh by electric spinning. As shown in Fig. 5a, PVDF solution with different mass fraction was in the needle tube, and PVDF solution was made into PVDF fibers by electrostatic spinning. A non-woven fabric with lower density was adopted as the substrate to receive the PVDF fibers. For the uniformly fabricated fibers, the averaged diameter is about 250 nm. Later, we made nanofiber meshes that were the sandwich structure out of non-woven fabric and PVDF nanofiber. Through change of mass fraction of PVDF solutions, we obtained corresponding nanofiber meshes with different density. The morphology of nanofiber meshes under different mass fraction PVDF solutions were shown in Fig. 5b. It could be observed that the densities of the fabricated fibers increase with increasing of mass fraction of solutions.

We further characterized the electrostatic effects of the fabricated sandwich structured PVDF nanofiber mesh. The open-circuit voltage was monitored here since the electrical resistances of both the nonwoven fabrics and nanofiber meshes were relatively high. By trial and error, the pyro- and piezoelectric effects of the fabricated nanofiber meshes were shown in Fig. 5c and d. The results demonstrated that the PVDF nanofiber with 11 wt% concentration outputted the highest open-circuit voltage, approaching 0.04 V at 362 Hz, as shown in Fig. 5c. The piezoelectric effects of the nanofiber meshes were shown in Fig. 5d, the concertation of 11 wt% also exhibited the highest open-circuit voltage, reaching 0.01 V at 100 °C. The similar pyro- and piezoelectric effects of the PVDF nanofiber meshes with the thin film may due to the pressure-generated certain degree of tension on the fiber to form β-phase during the electrostatic spinning process. The excellent pyro- and piezoelectric properties of the fabricated nanofiber meshes have the potential application in electrostatic filters, wearable electronic devices, or biosensors.

## Conclusions

In this study, the pyro- and piezoelectric PVDF films and meshes were successfully fabricated by mechanical stretching and electric spinning. The results showed that the stretched PVDF films exhibit obvious phase transition process, and hence inducing excellent pyro- and piezoelectric effects. Furthermore, nanofiber meshes received on a PP nonwoven substrate were also successfully fabricated by a simple electric spinning method, which exhibit relatively higher pyro- and piezoelectric effects by monitoring the open-circuit voltages. These properties could make it possible to be used as electrostatic filters, wearable electronic devices, or biosensors.

## Supplementary Information

**Additional file 1:** Figure S1–S6.

## Data Availability

The datasets used or analyzed during the current study are available from the corresponding author on reasonable request.

## Abbreviations

PLM: Polarized light microscopy
XRD: X-ray diffraction
FTIR: Fourier transform infrared spectrometer
Raman: Raman spectrometer
SEM: Scanning electron microscope
PVDF: Polyvinylidene fluoride
DMF: N,N-Dimethylformamide
