# Supplementary material for: Studies on the electrostatic effects of stretched PVDF films and nanofibers
Source: Nanoscale Res Lett. 2021 May 3;16:79. doi: 10.1186/s11671-021-03536-9 (PMC8093351; doi:10.1186/s11671-021-03536-9)
Supplement: Supplementary file 1 — Additional file 1: Figure S1–S6. [file 11671_2021_3536_MOESM1_ESM.docx]

**Supplementary Information**

**Studies on the electrostatic effects of stretched PVDF films and nanofibers**

Yixuan Lin^1^, Yuqiong Zhang^1^, Fan Zhang^1^, Meining Zhang^1^, Dalong Li^2,3^, Gaofeng Deng^4^, Li Guan^1*^, Mingdong Dong^3*^

^1^Department of Chemistry, Renmin University of China, Beijing 100872, P.R. China

^2^School of Marine Science and Technology, Harbin Institute of Technology at Weihai, Weihai 264209, Shandong, PR China

^3^Sino-Danish Center for Education and Research (SDC), interdisciplinary Nanoscience Center (iNANO), Aarhus University, Aarhus C, Dk-8000, Denmark

^4^State Key Laboratory of Building Safety and Environment，China Academy of Building Research，Beijing 100013, P.R. China

Correspondence: guanl@ruc.edu.cn (G.L.); dong@inano.au.dk (M.D.)

E-mail addresses for all authors: 2018102280lyx@ruc.edu.cn (Y. L.); zhangyuqiong@ruc.edu.cn (Y. Z.); zhangchem@ruc.edu.cn (F. Z.); mnzhang@ruc.edu.cn (M. Z.); lidalong@hit.edu.cn (D. L.); denggaofeng@cabrtech.com (G. D.)

**Fig. S1.** The thickness of PVDF films measured by a stair meter.

**Fig. S2.** The β-phase content of PVDF films with different concerntrations.


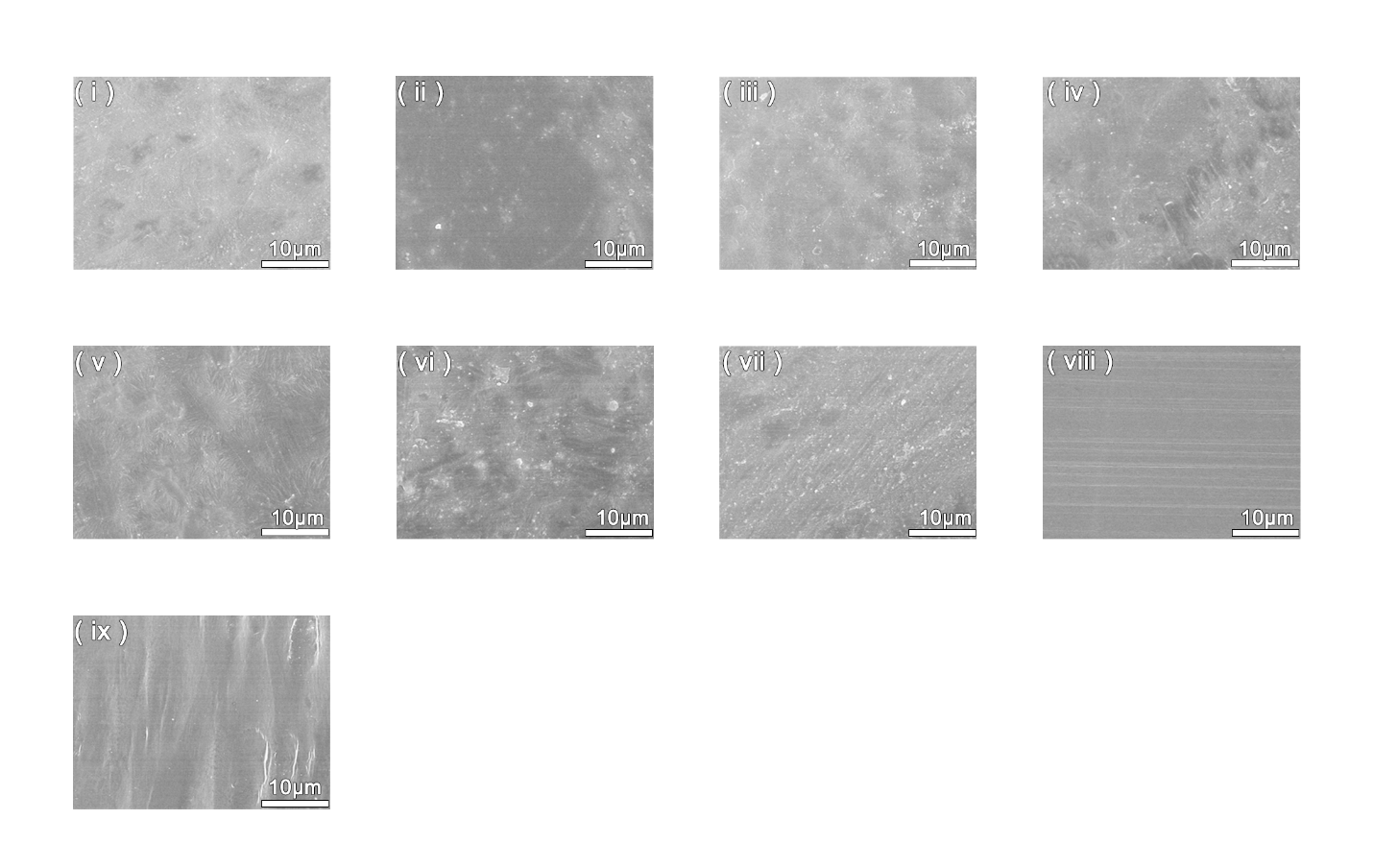


**Fig. S3.** SEM images of PVDF films with different stretching ratios. λ=1 (**i**),λ=1.02 (**ii**), λ=1.04 (**iii**), λ=1.06 (**iv**), λ=1.08 (**v**), λ=1.1 (v**i**), λ=1.2 (**vii**), λ=1.3 (v**iii**), λ=1.4 (**ix**).

**Fig. S4.** The crystal phase structure of PVDF films with different stretching ratios are characterized by X-ray diffraction.

**Fig. S5.** The DSC of PVDF films under different stretching ratios.


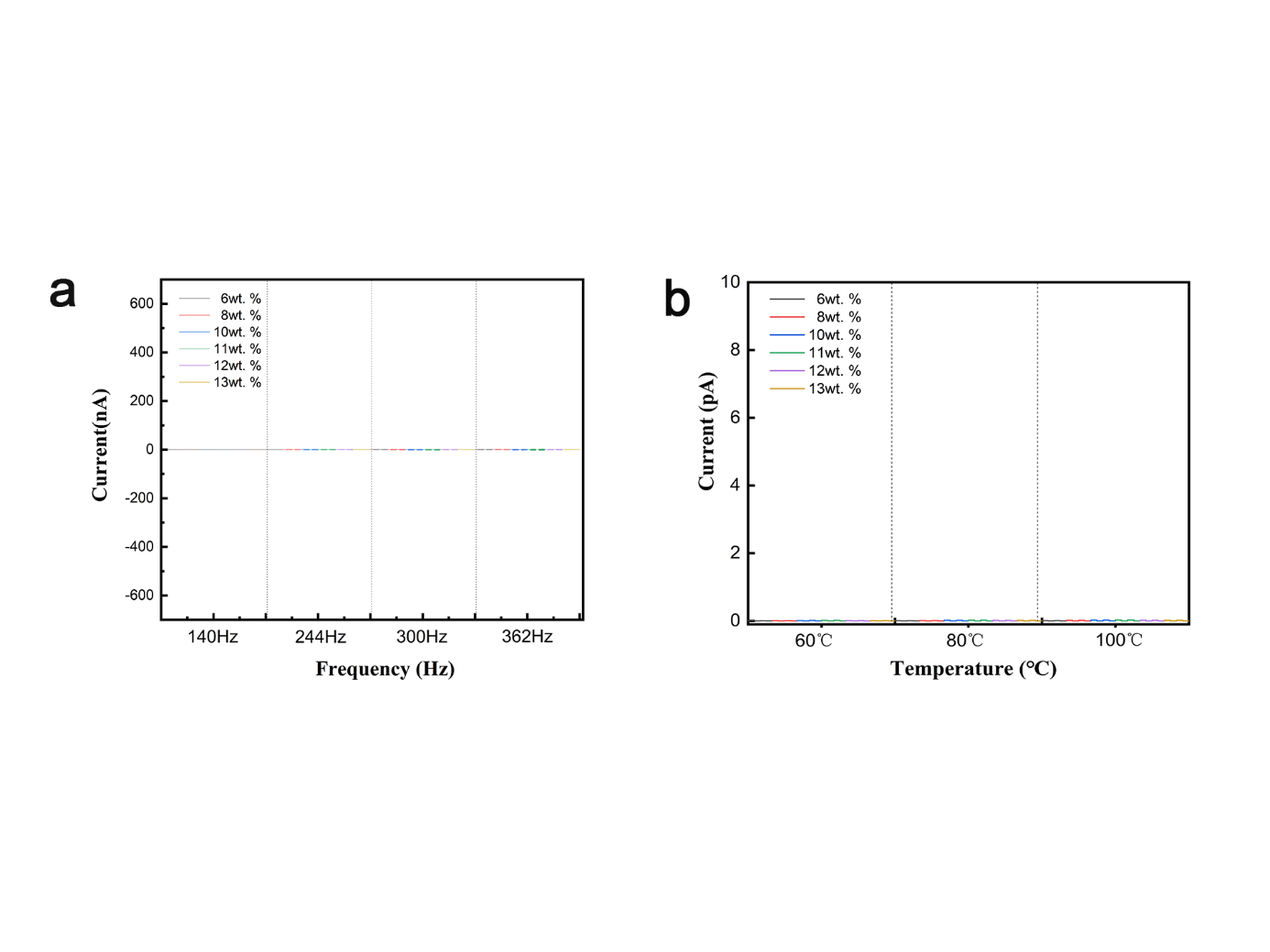


**Fig. S6.** The pyro- and piezoelectric effects of PVDF films before stretching. The piezoelectric effect of PVDF films before stretching. (**a**), the pyroelectric effect in PVDF films before stretching. (**b**)
